# P-1986. Prospective Evaluation of the Diasorin Simplexa Direct Assay for Molecular Detection of Candida auris Colonization in Patients

**DOI:** 10.1093/ofid/ofaf695.2153

**Published:** 2026-01-11

**Authors:** Ayesha Khan, Julie A Shimabukuro, Sidney Huang, Cassiana E Bittencourt

**Affiliations:** University of California-Irvine Health; University of California, Irvine Health, Orange, California; UC Irvine, Orange, California; University of California, Irvine Health, Orange, California

## Abstract

**Background:**

*C. auris* is an emerging drug-resistant yeast that is spreading rapidly in healthcare settings. High risk patients colonized with *C. auris* can later develop invasive infections with few to no treatment options. This underscores the importance of rapid and accurate detection of *C. auris* colonization for optimal infection control and patient management. The CDC recommends PCR over culture for screening. Here, we evaluate the first FDA-approved molecular assay, the Diasorin Simplexa ® Direct, which detects *C. auris* in patient screening swabs.

**Methods:**

We prospectively collected 891 composite axilla and groin swabs (BD, Copan ESwabs) from patients suspected of *C. auris* colonization or exposure. Performance of the Diasorin Simplexa ® Direct Assay was compared to: 1) *C. auris* culture (Remel CHROMAgar + MALDI) and 2) a standard of care RT-PCR, laboratory-developed test (LDT). Given that PCR tests are potentially more sensitive than culture, we also evaluated the combined performance of the Simplexa assay where the reference comparator value for each sample is the mode of the results yielded by the 3 methods (culture, LDT, Simplexa).

**Results:**

All 88 culture-positive samples were positive by Simplexa with a mean cycle threshold (C_T_) of 22.1 ± 4.4. Of the 803 culture-negative samples, 34 were positive by Simplexa with a mean C_T_ of 30.06 ± 5.5. Relative to culture, the Simplexa Direct yielded 100% sensitivity and 95.8% specificity. When the reference value for each sample was the mode of the results from all 3 study methods, the Simplexa yields a combined sensitivity of 100% (113/113) and a specificity of 98.8% (769/778). Of the 34 culture-negative/ Simplexa-positive samples, 25 were also positive by the LDT.

**Conclusion:**

The Diasorin Simplexa Direct is a rapid (∼2 h) and accurate FDA-cleared molecular assay to detect *C. auris* colonization in composite axilla and groin swabs from patients. It is practical and relatively easy to deploy in clinical laboratories relative to LDTs. The Simplexa likely has higher analytical sensitivity (lower limit of detection) than culture. This is supported by the relatively low mean C_T_ of 30 for culture-negative/ Simplexa-positive samples compared to a mean C_T_ of 22 for culture-positive samples, suggesting that the Simplexa Direct RT-PCR likely detected viable organisms.

**Disclosures:**

Cassiana E. Bittencourt, MD, DiaSorin: Grant/Research Support

